# Experimental dataset of U-folded falling chain under various openings with high-speed imaging

**DOI:** 10.1016/j.dib.2023.108913

**Published:** 2023-01-18

**Authors:** Athanasios A. Markou, Djebar Baroudi, Qiang Cheng, Hadi Bordbar

**Affiliations:** a Department of Civil Engineering, Aalto University, Rakentajanaukio 4A, Espoo 00076, Finland; b Department of Mechanical Engineering, Aalto University, Otakaari 4, Espoo 00076, Finland

**Keywords:** U-folded falling chain, Chain dynamics, Variable-mass dynamics, Whip cracking, High-speed imaging

## Abstract

A folded chain hanged by its two ends in U-shape at the same level with an opening, distance, between its two tips is known as U-folded chain, as studied in Markou et al. (2023). When one of the tips is fixed and the other one is released, the free tip of the U-folded falling chain accelerates faster than gravity, due to momentum conservation. This counterintuitive fact has long excited mechanicians around the globe. In the current paper we present a group of datasets (tip's coordinate timeseries), comprising three different subsets of this variable-mass dynamical system. A series of experiments of a U-folded falling chain of length 1.51m with total mass 31 gm, have been recorded with a high-speed imaging (2000fps) (Markou et al., 2023). The distance between the tips of the chain (opening) varies throughout the experimental session series: (i) 0.045m, (ii) 0.087m and (iii) 0.128m. Three timeseries of the tip's coordinates (x,y) have been extracted using an edge detector based method, from the recorded high-speed videos.

**Specifications Table**

**Table:** 

Subject:	Engineering
Specific subject area:	We are dealing with dynamics of variable-mass systems, which include a wide variety of fields from rocket theory [2], to biology [3] and economics [4].
Type of data:	Table Image Graph Figure
How the data were acquired:	A high-speed color camera (Photron Fastcam SA-Z [5]) equipped with a Nikon lens (Nikon AF Nikkor180mm f/2.8), which has the ability to capture high-resolution digital images at ultra-high speed, was used to record three video datasets. The post-processing of the images and the extraction of tip's coordinates as timeseries, has been implemented according to the work of Trujillo-Pino et al. [6].
Data format:	Analyzed (tip's coordinate timeseries)
Description of data collection:	A metallic ball chain was used for the experiments as shown in Fig. 1. The experimental set-up is shown in Fig. 2, where, one end of the chain, point O, is mounted rigidly, while the other one, i.e. point P in Fig. 1, is connected with a very thin string to a rigid support. To initiate the falling of the folded chain, the string was burnt.
Data source location:	• Institution: Aalto University • City/Town/Region: Espoo • Country: Finland
Data accessibility:	Repository name: Mendeley Data Data identification number: 10.17632/7vpt86xs85.4 Direct URL to data: https://data.mendeley.com/datasets/7vpt86xs85/4
Related research article:	Unfolding the dynamics of free-falling folded chain: experiments and simulations, Athanasios A. Markou, Djebar Baroudi, Qiang Cheng, Hadi Bordbar, International Journal of Non-linear Mechanics, 148, https://doi.org/10.1016/j.ijnonlinmec.2022.104257

## Value of the Data

• The data are valuable because they can be used to study variable-mass dynamical systems. Can be used to develop models that can capture experimental behavior.
• The data can be useful to physics, engineers, mechanicians that are studying phenomena, that occur in variable-mass dynamical systems.
• The data can be used as a validation tool for development of mechanical models that can capture the experimental behavior.

## 1 Objective

In order to develop reasonable models for the behavior of slender 1D structural elements under dynamic loading, a benchmark set of tests is required. To this end, the datasets presented in the current work, are used to calibrate models that can be used to describe the behavior of systems such as cables for example. In addition, these kinds of models apart from predicting the dynamic behavior of slender structural elements, they can also be used to describe and uncover physical phenomena, such as the chain fountain, [7]. Finally, the experiments are used to reveal physical phenomena behind the dynamics of variable-mass systems in general.

## 2 Data Description

**Fig. 1:**
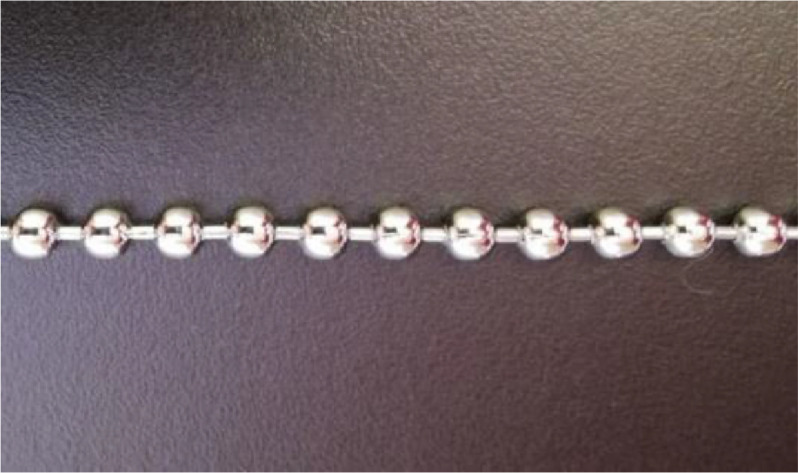
Ball chain.

**Fig. 2:**
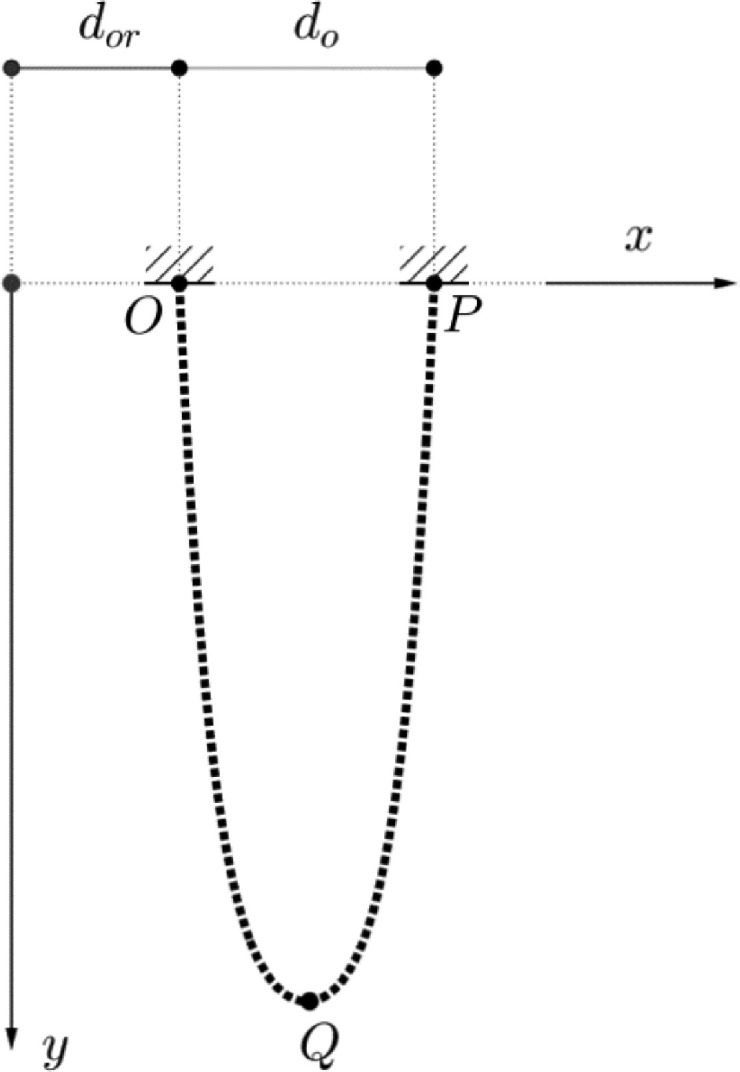
U-folded chain, where OPQ is the chain, *d_o_* is the opening and *d_or_* is the distance from the origin, modified from [1].

In Table 1, the physical parameters of the tested falling chain are presented. The ball chain used in the study is demonstrated in Fig. 1, while its maximum bending angles is 45 degrees. In Table 2, the opening d_o_ for the three different tests is shown. The data of the U-folded falling chain include the extracted tip's coordinate timeseries for the three different openings d_o_ (0.045m, 0.087m, 0.128m) between the two tips in its initial state (see Fig. 2).

**Table 1:** Parameters of chain experiment.

L (m)	M (g)	Diameter of ball (mm)	Size of link between balls (mm)	g (m/s^2^)	Air density (kg/m^3^)
1.512	31	3	2	9.819165133	1.2

**Table 2:** Test name with corresponding opening d_o_ and distance from origin d_or, see Fig.2._

Test	T2	T4	T5
d_o_ (m)	0.045	0.087	0.128
d_or_ (m)	0.242	0.312	0.310

The data contains txt files of the three different tests in txt files. All three txt files contain three columns of data: the first one is the time in seconds, the second one is the x-coordinate in pixels (px) of the tip and in the third one is the y-coordinate in pixels (px). The ratio between distance and pixel is given by 1.576482830 mm/px. The file “T2_TXY.txt” contains the data of test T2 (Table 2), the file “T4_TXY.txt” the data of test T4 (Table 2), and the file “T5_TXY.txt” the data of test T5 (Table 2), [8].

## 3 Experimental Design, Materials and Methods

The experimental sessions took place in the laboratory of School of Engineering in Aalto University in Espoo, Finland. The chain that was used during the experimental sessions is shown in Fig. 1 and its parameters are shown in Table 1. The one end the chain's tip is fixed, while the other one in hanged by a very thin string. In order to release the tip of the chain the string is burnt in an attempt to introduce zero initial velocity into the system. A high-speed color camera (Photron Fastcam SA-Z [5]) was used to capture the dynamics of the folded chain. The camera provides high resolution imaging with 1-megapixel CMOS image sensor with a full resolution of 1024 × 1024 pixels at 2000 fps. In the presented experiments, 2000 fps with an exposure time of 0.5 ms was selected. The number of frames recorded is 10000 for a record duration of 5 s. The scale factor between distance in millimeters and pixel is 1. 576482830 mm/pixel. In addition, a black curtain was inserted behind the U-folded chain to avoid the random noise from the environment, so that the postprocessing will be smoother. Finally, two halogen lights were used to provide homogeneous light along during the recording.

It is worth highlighting as well the uncertainties that were present during the experiment sessions. There are three groups of uncertainties related to: (i) experimental session, (ii) recording device and (iii) postprocessing of the recording data.

The uncertainties regarding the experimental sessions are related to: a) zero initial velocity during the release of the chain, b) 2D motion of the chain perpendicular to the camera, c) fixation point (point O, see Fig. 2) and its rigidity and dissipation ability and d) the sharpness of the lighting.

The uncertainties regarding the recording device are related to its limitations of the recording of the frames. The speed of the chain is higher in the falling phase compared to the rising one. To avoid loss of details the frame rate needs to be proceeded. Alternatively, use of higher recording frame rate results in larger required storing memory. An adjusted frame speed is used that tries to compromise between the loss of details and the large recorded file.

Lastly, the uncertainties regarding the post-processing of the data to extract the coordinates of the chain's tip are highly linked with the selected algorithm, [6]. More specifically, the accuracy of the edge detecting depends on the light intensity and the focusing ability. A blurry image can be produced under nonhomogeneous light and inaccurate focusing. In this dataset, the size of the chain's edge is around 1-2 pixels and this corresponds to uncertainty regarding the tip's location from 1.58mm to 3.16mm.

## Ethics Statements

The work did not involve human subjects, animal experiments or data from social media platforms.

## CRediT authorship contribution statement

**Athanasios A. Markou:** Conceptualization, Methodology, Writing – original draft, Software, Writing – review & editing, Validation, Investigation, Data curation, Visualization, Supervision, Project administration, Formal analysis. **Djebar Baroudi:** Conceptualization, Methodology, Writing – original draft, Software, Writing – review & editing, Investigation, Project administration, Formal analysis. **Qiang Cheng:** Data curation, Resources, Validation, Investigation, Software, Formal analysis. **Hadi Bordbar:** Writing – original draft, Supervision, Visualization.

## Declaration of Competing Interest

The authors declare that they have no known competing financial interests or personal relationships that could have appeared to influence the work reported in this paper.

## Data Availability

U-folded falling chain (Original data) (Mendeley Data).
